# Child Weight Growth Chart and Its Associated Factors in Birth Cohort of Maku Using a Growth Curve Model and LMS Method

**DOI:** 10.5539/gjhs.v7n6p181

**Published:** 2015-04-15

**Authors:** Seyyed Mohammad Taghi Ayatollahi, Zahra Sharafi, Elham Haem

**Affiliations:** 1 Department of Biostatistics, Shiraz University of Medical Sciences school of Medicine, Shiraz, Iran

**Keywords:** Infant weight rate, Infant weight chart, growth curve model, LMS method

## Abstract

**Background:**

Infant growth is defined as a positive change in body size over a period of time, and is a sensitive predictor of social health. The most effective way to determine child growth is by measuring birth weight and constructing a weight growth trajectory. Many studies were conducted on the effects of different factors on birth weight, but investigations of these effects on growth trajectory are really sparse. This study analyzes longitudinal data to determine factors affecting growth trajectory and used LMS chart for comparing children.

**Materials and Methods:**

In a cohort study, 256 neonates born in 2004 in Maku, Iran, were recruited and were followed until 2009.The weight of the neonates were measured at 12 occasions from birth, until the age of 5 years. A growth curve model was used to determine the affecting factors. The parametric LMS method was used to construct the reference centiles curve of the weight (5th, 50th, 95th percentiles).

**Findings:**

The findings show that while controlling the other factors, birth region, breast feeding duration, mother’s education and infants’ gender significantly influenced the longitudinal weight rate. However, other variables did not reveal any significant association with growth. The growth charts increased rapidly from birth to 5 years of age for both sexes. It was observed that male children grew faster than females, through the first year of age up to 5 years.

**Conclusion:**

Although every child has a growth potential, this capacity could be influenced by various factors and can be compared with other infants through a growth chart. We used longitudinal data to obtain the risk factor of growth trajectory. LMS method was also used for description of growth. Thereafter, the weight chart of Shiraz, southern Iran’s corresponding infants, was compared.

## 1. Introduction

Infant growth is defined as a positive change in body size over a period of time, and it is a sensitive predictor of an individuals’ health (Ayatollahi & Ahmadi, 2001; Ayatollahi & Shahsawary, 2002). An inappropriate growth pattern indicates nutritional disorders, chronic and infectious diseases in infants. Early growth rate is related to adulthood health (Ayatollahi & Bagheri, 2010; Ayatollahi, Bagheri & Heydari, 2013; Hosseini, Maracy, Sarrafzade & Kelishadi, 2014), such that it is important to develop the concept of appropriate growth for parents, in other to detect children with abnormal growth rates, as soon as possible (Hosseini et al., 2014).Weight and height of preschool children are the most common anthropometric measurements, to investigate a child’s growth rate (Ayatollahi & Bagheri, 2010; Ayatollahi et al., 2013; Fok et al., 2003). Weight is the simplest and most reliable anthropometric measurement (Kalies et al., 2005), and may be used as an indicator of childhood infection (Ayatollahi & Bagheri, 2010; Ayatollahi et al., 2013; Stephensen, 1999). On the other hand, height summarizes various measures of nutritional conditions in the first two decades of a human’s life (Inwood & Roberts, 2010).

Conversely, many researchers have performed cross-sectional studies to evaluate the birth weight of infants and growth, but longitudinal studies are very limited even in the developed world (Ayatollahi, 2004). Longitudinal studies are more useful compared to cross-sectional ones, because in the former, the development of subjects over time can be studied and used to assess the individual (Hosseini et al., 2014). Also in growth studies, the weight of children were measured repeatedly over time, these measurements are correlated. Moreover, correctly accounting for correlation is crucial for estimating parameters and testing hypotheses. Several longitudinal growth studies have been carried out in Iran since 1966 (Amirhakimi, 1974; Ayatollahi & Ahmadi, 2001; Ayatollahi & Shahsawary, 2002; Ayatollahi, 2005; Ayatollahi, 1993; Ayatollahi & Rafiee, 2006). Most of these studies have presented growth standard percentiles and are preferred for local standards (Ayatollahi & Ahmadi, 2001; Ayatollahi & Rafiee, 2006). However, few of them attempted to investigate the affecting factors using a growth curve model. This model is a special case of mixed effect models that is appropriate in longitudinal data, for finding factors that affect the longitudinal response.

Therefore, the aim of this study was to investigate the effect of different factors on the weight growth trajectory of preschool children of Maku birth cohort, who were followed longitudinally from birth to 5 years of age, using growth curve modeling. We also intended to present the growth weight of the birth cohort, and compare the relevant measures with that of Shiraz’s study.

## 2. Materials and Methods

Maku, a city in West Azerbaijan, is located in the northwest of Iran and borders Turkey. It enjoys a semi-arid and cold climate with a population of about 200,000 including 8.1% of the total population of the province. A birth cohort of 256 neonates (124 boys and 132 girls) without apparent congenital anomaly born in Maku and its village in 2004, were recruited randomly using a multi-stage random sampling procedure from all existing maternity clinics. The subjects were followed for five years until 2009 and visited at the health centers periodically. A questionnaire on demographic and health status of the neonates and their parents including anthropometric measurements was completed. The subjects’ weights were measured by a trained auxologists at birth*; one, two, four, six, nine months and 1, 1.5, 2, 3, 4 and 5 years of age. Weight was measured in light clothing to the nearest 10 g until the second year of age, using baby scale and onwards to 0.1 kg.

The effect of parents’ age, education and occupation, child rank, age of using supplement food, breast feeding duration, gender and region (city or village) was studied in this survey.

However, the result of this study was compared with 317 Shirazi infants (164 girls and 152 boys) who were selected randomly among those born at the 14 maternity clinics of Shiraz in 1996 and followed for two years. Their weights were measured at birth, 1. 5, 3, 4.5, 6, 8, 10, 12, 15, 18, 21 and 24 months (Ayatollahi, 2001).

### 2.1 Statistical Analysis

In the present study, we used a growth curve model to analyze our longitudinal data. Growth curve model allows analysis of longitudinal or repeated measure data sets, where individuals may have missing measurements on one or more occasions. The growth curve model was proposed as:


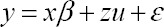


Which is a special case of mixed effects models. In this model, y depicts a growth index (weight), x is a matrix of fixed effect variables (parents age, education and occupation, child rank, age of using supplement food, breast feeding duration, gender and region (city or village)), z denotes matrix of random effects variable (intercept and Infants’ age), while β and u are parameters of fixed and random effects, respectively. Parameters were estimated using restricted maximum likelihood (Bliese & Ployhart, 2002).

Furthermore, the LMS method (Pan & Cole, 1997) was applied to calculate smoothed weight centiles. The LMS method describes the changing distribution by the median, the coefficient of variation and the skewness of distribution with M, L and S curves. We used a penalized likelihood to fit the L, M and S curves by non-linear regression as cubic splines, and the extent of smoothing required can be expressed in terms of smoothing parameters or equivalent degree of freedom. We also tested the normality of data by the Detrended Q-Q plot. Z score diagram was used for monitoring the outlier data.

SPSS version 16 (SPSS Inc., Chicago, USA) was used to analyze the general aspects of the data. Growth curve model was performed using SAS 9.2 and was used to determine the influential factors. LMS chart maker and Excel softwares were used for construction of centile

## 3. Results

Almost all mothers were housewives [247 (96.5%)], of whom about three fourth delivered their babies normally [189 (73.8%)] and the rest by cesarean section [67(26.2%)]. Over four fifth of the mothers stated that they had planned pregnancy [221(86.3%)]. Illiteracy rate in fathers and mothers were 12.9 and 26.6%, respectively. As shown in Table 1, mothers’ education was considered in three categories: elementary (0-6), high school (6-12) and university, and breastfeeding duration categories in less than six, six to twelve and more than twelve month.

**Table 1:** Summary of demographic discrete variables

variable		N (%)	variable		N (%)
Mother education	0-6	158(61.7)	Infant sex	boy	124(48.4)
	6-12	90(35.2)		girl	132(51.6)
	>12 years	8(3.1)	Breast feeding duration	0-6	20(7.8)
Region	city	156(60.9)		6-12	26(10.2)
	village	100(39.1)		>12 months	210(82)

The intercept variance and the age slope variance are significant (p<0.0001), suggesting that a model that allowed age as random, would be better than the model considering age as fixed. Table 2 shows the effects of infants’ weight using a growth curve model. Infants’ age was highly significant when the effect of age was random. Gender (P<0.0001), birth region (P=0.003), breast feeding duration and mothers’ education had a significant effect on weight growth trend. Infants breastfed for less than 6 months had a lower weight in comparison with their counterparts. Also, infants of educated mothers had significantly higher weight. Other fixed effects covariates were not significant (p>0.05).

**Table 2:** Effects of infants’ weight risk factors using growth curve model

variable		estimate	SD	p-value
Intercept		1.09	0.1	<0.0001
Infants age		1.25	0.01	<0.0001
Mother education	0-6	-	-	-
	6-12	0.5	0.1	0.04
	>12years	0.72	0.2	0.03
Breast feeding duration	0-6	-	-	-
	6-12	0.35	0.18	0.05
	>12months	037	0.14	0.01
Infant sex	Boy	0.33	0.07	<0.0001
	Girl	-	-	-
region	City	0.24	0.08	0.003
	Village	-	-	-

**Figure 1:**
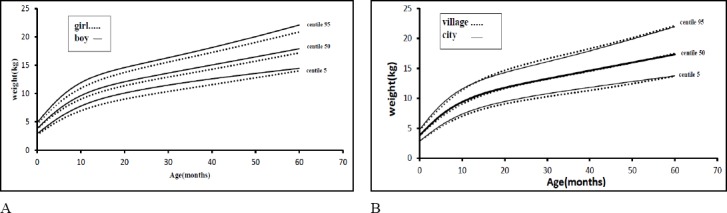
Weight chart of infants in Maku, Iran: (A) Weight chart by gender solid line implies boy curve and dash line implies girls. (B) Weight chart by birth region solid line implies city curve and dash line implies village

Figure 1A shows the weight charts for both sexes between the ages of 1 and 60 months for weight. These weight curves indicate higher weight in boys than girls and this difference is the same from the time of birth until 5 years of age. As shown in Figure 1B, rural infants had a lower weight in comparison with urban ones but this was negligible. Sample size is not sufficient for constructing weight for age centiles for different group of covariates.

**Figure 2:**
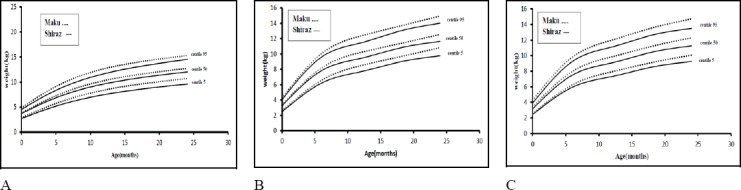
Weight chart of infants in Maku and Shiraz Iran; solid line implies Shiraz curve and dash line implies Maku (A) Girls (B) Boysc (C) city

In this study, we also compared the weight curve of Maku with Shirazi infants (Ayatollahi, 2005) by LMS method. Figures 2A and 2B compared the median and extreme centiles of the weight of Maku and Shiraz infants by sex. We also compared urban infants of Maku with Shirazi infants, as shown in Figure 2C.

## 4. Discussion

We used growth curve model analysis of growth data from a large prospective birth cohort and observed the marked effects of sex, mother education, birth region and birth feeding duration. In most of the previous studies, the identification of factors was carried out via repeated measures and centile statistics. But growth curve modeling allows analysis of unbalanced repeated measures and longitudinal data for this purpose (Cnaan, Laird, & Slasor, 1997).

In this study, gender had a significant effect on weight growth trend, such that boys were significantly weightier than girls from birth up to 5years of age. In the studies of Hosseini et al. (2014) and Ong et al. (2002), boys were weightier than girls from birth up to 2 years of age (Hosseini et al., 2014; Ong, Preece, Emmett, Ahmed & Dunger, 2002). This could reflect the influence of the Y chromosome gene in producing testosterone in both fetal life and during the first few postnatal months. The increasing size and growth rate of the male might be due to production of testosterone (Smith et al., 1976). This finding is in contrast to that of a study in Jahrom (Iran), indicating that boys were heavier than girls but the mean weight difference between them was not statistically significant (Heydari, Emamghoreishi, & Amini, 2005).

Mother education had a significant effect on weight growth trend, such that infants of educated mothers were heavier than their counterparts. In Hosseini et al.’s study, the birth weight of low educated mothers was significantly lower than the children of mothers with higher education (Hosseini et al., 2014). However, in Ong et al.’s study, mother’s educational achievement did not affect weight at birth (Ong et al., 2002).

Many studies documented that child’s rank had a significant effect on the birth weight and weight growth trend. In a study by Hosseini et al., firstborn children were smaller and thinner than their counterparts, from birth up to 2 years of age (Hosseini et al., 2014) while in Ong et al.’s study, firstborn infants were smaller and thinner at birth but showed a dramatic catch-up in weight, so they overcompensated their initial size deficit and the result was larger childhood size (Ong et al., 2002). In this study, the birth rank had no significant effect on weight growth rate.

Parental age and occupation did not have a significant effect on the child’s weight growth trend. This finding is in line with those of Hosseini et al. In a study by Raum et al. (2010), mother’s age had no significant effect on child obesity risk in 6-year-old children (Raum et al., 2011).

Further, the present study shows that infants breastfed for a longer duration had a significantly higher mean weight, up to 5 years of age. Meta-analyses in 2005, showed an inverse association between breastfeeding and risk of obesity at different ages (Harder, Bergmann, Kallischnigg & Plagemann, 2005). However, a study in 2012 revealed a non-significant relationship between breastfeeding duration and overweight (Vafa, Moslehi, Afshari, Hossini & Eshraghian, 2012).

Weight standards in the Maku infants were slightly higher than those of Shirazi infants for both sex and urban region. This may be due to ethnic or weather differences between Shiraz and Maku. Shiraz is in the southwest of Iran and Maku is in the northwest, having different weather and ethnic groups.

Fundamentally, a sophisticated statistical method (growth curve modeling) to assess the association between various environmental factors and response variables was performed in this study but growth could be affected by genetic, nutritional, hormonal and other environmental factors (e.g. socioeconomic status, level of activity, child caring, etc). The authors suggest that these factors be analyzed in future studies. Future studies can also report weight for height or weight for length instead of weight for age because this also is an appropriate measure during infancy. It is suggested that one should be cautious about the much needed samples, in order to report LMS chart in subgroups of the interested factors.

In conclusion, we presented growth weight chart in North East Iranian infants by analyzing longitudinal data with the parametric LMS method. Also, the factors affecting growth weight that is useful in policy making for the development of individual health in the society were determined.
